# The Territorial Distribution of the Population for Purposes of Sanitary Inquiry and Social Economy

**Published:** 1856-10

**Authors:** Henry Wyldbore Rumsey


### ON THE TERRITORIAL DISTRIBUTION OF THE POPULATION, FOR PURPOSES OF SANITARY INQUIRY AND SOCIAL ECONOMY.

By HENRY WYLDBORE RUMSEY, Esq.

[*Read before the British Association for the Advancement of Science. Cheltenham, Aug.* 12, 1856. *Section F.*]

In the present condition of society, but few and rare opportunities are afforded to States to group their populations on scientific principles, to determine the most salutary and beneficial sites for human habitation, or to combine the sites so occupied in well-contrived districts for the purposes of statistical inquiry and local management. No reasonable excuse can be made for omitting to provide for a judicious allocation and distribution of a *new* population, as in the case of military colonies, and of settlements on unoccupied tracts of land. To neglect this public duty must be to inflict irreparable injury not only upon the first occupants, but even more seriously upon their descendants; as has been unhappily proved by numerous instances of unskilful migration and colonisation in the history of our own race.

Enough is already known of the influence of climate and soil, and other natural features of locality, upon man's physical and psychical condition to prevent any gross mistakes being made in future, either by governments or by the promoters of voluntary and associated enterprise.

We are now in a position to show that no occupation of places, notoriously and obviously unhealthy, for mere commercial purposes, is justifiable, or likely to be ultimately beneficial to a community. How great the peril and loss which have resulted from peopling such spots as the deltas and *embouchures* of large rivers; the banks of sluggish streams; alluvial soil periodically deposited by floods upon lands where drainage is most difficult, if not impossible; closely pent-up valleys among mountain ranges and in clefts of table land, tempting the ignorant and unsuspicious by their beauty and verdure and apparent security! Surely, the consequent destruction of life (in some cases enormous); the deterioration and degradation of the inhabitants, not only in their physical structure, but in *their mental and moral excellence;* and the immense sacrifice of national and personal wealth which is afterwards demanded to palliate the frightful evils which man has thus inflicted upon himself; are considerations of sufficient importance to decide the national course for the future with regard to colonisation or migration.

2. But, in England, we have to deal with an *old* population, rapidly advancing in civilisation, having its established laws, its recognised divisions, its carefully protected rights of property (both corporate and private), and, moreover, extremely jealous of changes, even when proved to be conducive to the public welfare. Hence the difficulties which attend any re-opening of the question.

But these difficulties are not greater than many which the persevering Anglo-Saxon race, with its Scandinavian and Norman elements of force and energy, has often overcome.

I would rapidly glance at some of the causes of population movement which have occurred since the settlement of our island by its present composite race. In the middle ages, locomotion was common enough. If later facilities of transit were wanting, later obstacles to it had not then arisen. A hardy and daring people, of simple habits and few possessions, living in a country only partially enclosed, had little to check their migratory tendencies, except the will of chiefs and conquerors, where that could be enforced. Clans, serfs and villans were bound far more to their lords than to the land, and moved at command. Continuous practice in civil and foreign war enabled armed bodies to shift rapidly and easily from place to place, while settlements for industrial purposes were as repeatedly changed, to escape feudal oppression, military exaction, or religious persecution. Even the lonely and helpless travelled in those times more securely, if not comfortably, than some are disposed to think. The age had its decencies, as well as its asperities, and special means of protection existed. Inns, or rather hospitals (*hospitia*, whence our old "hospitality") abounded, and ecclesiastical refuges were thickly scattered throughout the land.

But after the fifteenth century, the gradual consolidation of the parochial system of England checked irregular migration and regular vagrancy; and it is needless to describe, for we all know, the effect of that system in *fixing* each man, woman, and child to a certain spot. The great social reform effected in the reign of Elizabeth led to the settlement laws, and the people became, in a peculiar sense, *adscripti glebæ*. Hence, as roads did not improve, and as property with its attendant comforts and conveniences increased, the parishioner became less inclined to move and more apprehensive of the perils of travel, while the man of substance made his will before a journey of fifty miles.

Now, however, the social immobility of the seventeenth and eighteenth centuries has again relaxed, and is fast passing away. The settlement laws have been greatly modified. We are again becoming, in some sort, a nomadic population. Business, speculation, science, health, pleasure, and the mere love of change (a kind of vagrancy) shift us from place to place, unsettle our local associations, and loosen our local bonds. The very character of our habitations is changing; the massive stones and timbers which formed the wall of one house, three or four centuries ago, would now suffice, under structural improvements and economy of material, for half-a-dozen houses. Iron and wooden buildings, of all sorts, sizes and shapes, fitted for all purposes, domestic and public, are now made and sold in great numbers, ready to be packed and transported, commodiously and cheaply, to any distance, by railway or ship. No Mongolian wanderers of Central Asia ever struck, carried and pitched their rude tents with such facility as we now do our skilfully constructed portable houses. The legal transfer of property is also becoming far less costly, and therefore more common. Another barrier to change of residence is thus removed.

3. But the most important movement of the population in modern times has reference to its local aggregations. To go back no further than the beginning of the present century; the inhabitants of cities and towns in England did not then number one-third of the total population; but during the half century ending in 1851, they had increased so rapidly as to equal the population of the rural districts.

In connexion with this greater compression and condensation of the population in certain localities, many and serious evils—physical, social and moral evils—which I need not here specify, have accrued; but the results are beginning to be so much more clearly felt and understood by the people themselves, that a movement of a corrective and compensating kind has lately arisen, and is now, I believe, proceeding at a considerable pace. The crammed interiors of towns are gradually disgorging the human masses which they have swallowed (it may be said) too quickly for a safe social assimilation. New openings and lines of street, through the centres of closely built cities, are dispersing their banefully crowded inhabitants. Narrow lanes of tall houses, confined courts and alleys, dark basements and cellar dwellings, reeking with all physical and moral impurity, are now being emptied of their injured occupants; even dwelling-houses of a better sort in the heart of old towns are being fast converted into warehouses and workshops, so that a steadily diminishing proportion of the people sleeps within ancient municipal limits.

The working classes, thus displaced, are finding accommodation in the outskirts, and spreading over the surrounding districts. Thus, the benefits of an almost rural residence are no longer exclusively enjoyed by the man of office, the banker, the lawyer, and the merchant; for the small tradesman and the clerk have followed the wholesome example, and the centrifugal impulse now extends even to the artizan and the porter.

Suburban areas are accordingly increasing much faster than urban populations, while the universal railway enables a far larger proportion of the working classes to live conveniently and economically, beyond the reach of town smoke, to see the sun rise, and to breathe the fresh air of green fields.

4. This movement, be it observed, is advancing in the teeth of laws and institutions, which are now worse than useless. No one would pretend that the original object of municipal organisation any longer influences our population. We need no protection against monarchical or feudal tyranny. Yet the tendency of all past legislation, even to this day, has been to impose narrow territorial limits upon town communities, and to tempt the people into injurious aggregation.* To abolish our absurd and mischievous isolations of political rights, and to extend municipal privileges to the entire population of the kingdom, would materially aid in restoring that salutary balance of town and country population which has been temporarily disturbed by the rapid advance of commerce and manufacture, unaccompanied by provisions for a beneficial allocation of the people.

But whatever may be done or left undone as to parliamentary representation, it is clear that the gradual outpour-

* Certain political privileges, to say nothing of the substantial advantages which it is found so difficult to separate from the parliamentary suffrage, have been exclusively bestowed upon the freemen and burgesses of *boraughs;* and the working man, in too many instances, has not feared to encounter the higher rent of a house within the limits consecrated by parliament, because he knows that, when his vote is wanted, his landlord will be paid. A wise reversal of such mischievous legislation might promote that most desirable and sanitary reflux of the population, to which I have already adverted. If owners of real property (freeholders) ought still to be represented by "knights of shires", why should not tenants in rural districts share that representation which is now possessed exclusively by tenants in boroughs? Is not the ten-pound house in the country (I do not say, its occupant) superior in every respect to the ten-pound house in the city? If a householding qualification for voters be still considered essential to the safety of the "British constitution" (?), why not confer upon every such householder, throughout the land, the right of voting in that borough with which he might be most conveniently connected? Why not thus enlarge the areas of parliamentary boroughs until their limits meet, and finally settle the question of future extension?

ing of town populations will remove many current objections to an amended division of the country for statistical and sanitary purposes; and we shall, therefore, do well to look closely and carefully into the anomalies and perplexities which attend upon the various systems of territorial distribution.

5. Our old parochial divisions are not uncommonly found to be wholly irreconcileable with the altered grouping of the people. If the venerable parish church, with its sacred and ancestral associations, still attracts, and, to a certain extent, localises the population in many rural districts,—the colossal mill, the palace of manufacture, the coal field, the mine, and the harbour, determine more rapidly and imperatively the aggregations of a commercial and industrial community,—and, I must add, too often with utter and appalling neglect of the physical and moral prospects of the population thus brought together.

These new hamlets and townships are almost necessarily formed without reference to parochial boundaries, which, if not revised and corrected to meet the changes of population, become practical nuisances. Hence, in populous districts, legal powers are occasionally conferred upon municipal or ecclesiastical authorities to alter and amend parochial limits. As new sites are peopled, new divisions and boundaries must be settled. But, I would ask, are these alterations made upon any definite and well considered principles? Does science aid them? does enlightened experience direct them?

Beside the parochial divisions of the country, there are others as ancient, yet still more in conflict with the changing localisation of the people. Tithings and hundreds have generally become little more than matters of history. Boroughs and cities very rarely include an exact number of parishes, or coincide precisely with parochial limits; parts of the same parish are commonly to be found on either side of the same municipal boundary, and even in different counties.*

6. But the comparatively recent division of the country into *Poor-Law Unions* was based more accurately upon the parochial system, and unions are generally exact aggregates of a number of parishes. The same union, however, often includes parishes or parts of parishes in two or three counties; and, generally speaking, no kind of relation exists

* The late Mr. Rickman noticed, that "there are in England and Wales about 550 parishes which are known to extend into two counties, or into more than one hundred, or other division." (Census of Great Britain, 1851, 8vo, p. 24.)

between the boundaries of boroughs and those of parochial unions. The latter were also formed without any regard to the natural features of the district. Great facts of physical geography were wholly ignored. The able and learned gentlemen who were officially employed to describe, define and form the unions, did not profess to be guided by scientific considerations, or to be influenced by established principles of hygiène. Science, commonly so called, had, therefore, no effect upon their decisions. It would even appear that facility of communication between different parts of the same union or district must have been purposely set aside in many instances. It was probably, in such cases, rather an object to place some highly pauperised group of population at a distance from the sources of relief on which it had learnt so injuriously to rely. The hilly ridge, the pathless morass, the bridgeless river, were therefore seldom considered as necessarily marking the limits to a union or district.

Without enumerating other divisions, ancient and modern, some for the administration of justice, others for taxation, others for ecclesiastical polity, I have already noticed enough of conflict and diversity among all these systems of partition to convince any one of the great difficulties under which both official recorders and students of vital statistics must labour.*

7. The registration system of this country, with which is now combined the machinery for the census, is based upon the Poor-Law division into unions. The registration districts are, therefore, 620, and the subdistricts 2,190 in number, each subdistrict containing, on the average, seven parishes, townships, or places, and some populous parishes being divided for the purpose. Thus, the returns of population, births, deaths, and marriages, in "union" districts, have made the defects of the Poor-Law division more obvious; while an erroneous distribution of the people has, in turn, affected the compilation of vital and sanitary statistics. Moreover, no information upon such matters is offi-

---

* "The inconveniences and perplexities which the variety of ecclesiastical, military and civil, fiscal and judicial, ancient and modern, municipal and parliamentary, subdivisions of the country occasion, have been sensibly felt by us, as they were brought under our notice in the enumeration of the population. It is not within our province to reduce all these to simplicity and harmony; but we call attention to their existence, and venture humbly to suggest that the task of taking any future census, the comparison of statistical facts of every kind, and probably all administrative arrangements, would be greatly facilitated by the adoption of an uniform system of territorial divisions in Great Britain." (Census of Great Britain in 1851, 8vo, p. 25.)

cially published in each locality.* So that it has been found extremely difficult to ascertain the bearing of any natural or artificial features of a particular tract of country, or any social characteristics of a populous district, upon the life, health, and welfare of its inhabitants.

To give an instance; the other day, I was questioned by one of the Vice-Presidents of this section concerning the physical and sanitary condition of the population of the Forest of Dean, in this county; a district of peculiar geological formation, bounded by important rivers, and inhabited by a very distinct class (I had almost said race) of people. But the fact came out, that the registration and census divisions give no collective information on the subject; for not only do those unions, which contain most of the Forest parishes, embrace other portions of population in widely different circumstances, but no fewer than twelve of the Forest parishes, containing a total population of more than 20,000, are contained in unions nominally belonging to the adjacent counties—Herefordshire and Monmouthshire. Nor does it appear that those Forest parishes, which are included in the adjacent "registration" counties, constitute separate districts, so as to admit of being again grouped with the Forest subdivisions, for statistical returns. To determine correctly the physical and social condition of this remarkable population would, therefore, require a completely new arrangement of the parochial groups, and a new compilation of ultimate facts.

I might give other instances within my own limited sphere of observation, especially in unions containing both town and country populations—the several districts of which have not been determined with reference either to the condition of the inhabitants, or the natural features of the inhabited surface—and from which, therefore, *no trustworthy statistical deductions can be drawn.* For this, among other valid reasons, it is unsafe to adopt the rate of mortality in any union (registration district) as a test of the actual salubrity either of its principal town or of its more scattered population. The apparent rate of mortality in large towns is swelled by deaths in hospitals and workhouses of people who lived in distant country parishes. Either the mortality of all such institutions should be returned separately from that of the districts in which they are contained, or the deaths should be carried to the account of the several parishes from whence

* Except in places where officers of health have been appointed, and there only partially.

the patients come. Periodical returns of the vital force of the population (*i.e.*, the ages of the living) are also essential to correct such conclusions as are drawn solely from the number of deaths. I must, therefore, repeat, that until our vital statistics are more complete, and are compiled from a more scientific classification of the people, we can arrive at no satisfactory conclusions respecting the life, the health, the social state, the education, the morals, and the habits of those who inhabit the several places; we are unable to demonstrate the causes of social evils; and, therefore, we cannot call upon the legislature to inaugurate those reforms which, in this country, can be carried into effect only by the cordial co-operation of the enlightened and influential portion of the community.

9. Another obstacle to a scientific division of the country arises from the coexistence of several sorts of local *administrative bodies*, exercising conflicting functions, and with different areas of jurisdiction.

On the one hand, we have boards of guardians, elected by ratepayers and owners of property, and aided by the magistrates of the county, as *ex officio* guardians. These boards, as was well shown before the Committee on Sir B. Hall's Bills in 1855, superintend the interests of the entire population, and have already so many sanitary functions to perform, that they cannot and (we may rely upon it) will not be set aside by any general measure of public health which would leave them out of the question. It was, therefore, in my opinion, a mistake in the last Act for the Removal of Nuisances, etc., to confer upon boards of guardians the "wooden spoon" distinction among the corporate bodies, a list of which is given in that Act, in the order of their assumed fitness for local sanitary administration.

On the other hand, we have, in most towns, either local boards of health, or bodies of town commissioners, or town councils, elected in various methods, some tolerably good, others open to objection, but none, as far as I know, superior to that adopted for the election of boards of guardians. And to these bodies another class of sanitary functions is committed, though not exceeding in importance those exercised by boards of guardians. There are, therefore, two kinds of functions, imperfectly defined, and exercised in most places by at least *two* elective bodies, with different areas of jurisdiction—the sanitary and vital statistics being collected by that body, which has been the most distrusted as regards the sanitary management of the district.

10. Before proceeding to practical suggestions, I would briefly call attention to some of the anomalies and inconveniences which have resulted from limiting the execution of sanitary powers to bodies representing only dense and circumscribed portions of the population.

Among the practical evils of limited jurisdiction, I may notice, (1) the difficulty of obtaining an adequate and unexceptionable water supply; (2) the difficulty of providing outlets for common sewers, and markets for the products of sewerage: (3) the difficulty of protecting streams and rivers from defilement and impediment; (4) the difficulty of securing suburban places of sufficient extent for public recreation, for the erection of sanative institutions, and for the burial of the dead.

Again, the population surrounding these confined jurisdictions is excluded from even that small amount of benefit which may be derived from existing local sanitary administration. In a report on the mortality of Gloucester, which I presented to the Registrar-General in 1848, I showed, by a comparison of the deaths, the ages at death, and the apparent causes of disease, in the city proper and in the suburbs, that the latter were by far the more unhealthy and the more urgently in need of vigorous measures of sanitary reform. Mr. Cresy, the superintending inspector sent by the General Board of Health, confirmed the correctness of my distinction, and accordingly recommended that a considerable district of country, extending in some directions two miles from the centre of the city, should be included within the jurisdiction of the local board. But political questions arose; the town council claimed exclusive powers, and the provisional order was ultimately applied only to the parliamentary borough. The result was thus described, four years afterwards, by a resident gentleman of great intelligence, and belonging to no profession:—

"The jurisdiction of the Local Board, I am sorry to say, does not extend beyond the boundaries fixed by the Municipal Act, which practically excludes one-third [more now] of the population from any control. Upon this serious obstruction to sanitary improvement, my attention has long been fixed. I counted seven hundred houses on one side of the city, whose only drainage is Sudbrook, all beyond control; and, owing to the direction of the prevailing winds, the town has the full benefit of all the effluvia which their refuse creates."

The suburban residents looked in vain to the managing authority of the outlying districts for redress; for, said he, "The board of guardians does not co-operate with either the Local or General Board, but, I believe, offers all the obstruction in its power."*

Now this I believe to be a very common case.

11. The suburban population of towns, for reasons already stated, consists more and more of persons belonging to the humbler classes of society; and unless efficient building laws, founded on established sanitary principles, be enacted and enforced to protect the working classes against the injurious speculations of unscrupulous capitalists; unless, moreover, a sounder system of education than the present be extended to the whole working population—I mean a moral, industrial and physiological education, which may enable men and women to comprehend their true relations to the external world, and their duties to society and to their families;—unless they are thus trained in thrifty habits, and induced to devote some portion of their wages (now often worse than wasted at the beer house, the gin shop, and the casino) to the payment of a somewhat higher rent for well located, well drained and well ventilated dwellings; unless, I say, these social changes be effected, *we must expect to see an aggravation of sanitary and social evils among the suburban and rural, as well as among the town, populations*.

12. Again, "the error of commencing sanitary legislation, by circumscribing sanitary jurisdictions, has led to the adoption of a canon of administration, wholly unreasonable and undefensible, namely, that all districts in which it cannot be proved that an excessive number of persons die annually shall be exempted from the operation of sanitary law.

"Observe the difficulties into which the movement party has brought itself by this concession. The first question of the objectors is—What do you mean by excess of mortality? All above *twenty-three* in a thousand of the population—the average of English mortality, according to the Public Health Act. All above *seventeen* in the thousand—the "natural rate" of mortality—pleaded our first vital statist. All above *twenty-seven* in a thousand replied the ingenious and indefatigable advocate of the parish-vestry party. All above *twenty-five* in a thousand, concluded Parliament, because that number split the difference between Sir Benjamin Hall and Mr. Toulmin Smith.

"The excess of mortality being thus summarily, if not satis-

---

* Essays on State Medicine, pp. 333-4.

factorily, settled,—the fatal effects of the want of a preventive law having been correctly calculated—the required number of lives having been prematurely sacrificed to the regulated neglect—the preventive law may then, and not till then, be enforced. One is irresistibly reminded of the stolen steed and the order to fasten the stable door. All this, be it observed, is based on the assumption that a certain annual ratio of deaths, in any spot, is *the* test of its insalubrity. To argue further on such a point would seem to be a mere waste of time. Yet I must be allowed, by way of illustration, to to ask—What would have been thought of a proposition to restrict the application of the new Poor-Law to parishes in which the rates exceeded so many shillings in the pound? Or, of a Bill to abolish the constabulary force in every county or district in which less than an average number of crimes were committed annually?"*

The several objections which I have now urged against existing divisions and isolations of the inhabited surface of this country, I beg to recommend earnestly to the consideration of this section; and I submit that the difficulties in the way of a revision in the census and registration division of the country, for sanitary purposes, are of no great magnitude, and certainly not insuperable.

13. My first practical deduction would be, that no system of territorial distribution of population deserves to be either defended or adopted which does not secure for every portion of the country, whether town or rural parish, the superintendence of a uniform administrative machinery, competent to collect *all returns relating to the numbers, the vital force, the mortality, the diseases, and the reproduction of the population*, as well as to carry into effect all sanitary precautions. Now, in order to provide this general benefit, the boundaries of the registration districts ought to be revised with reference to physical topography, and the subdistricts, especially, should be so contrived, that, if possible, each may contain a population under the same physical circumstances,† while its shape and extent should be such as to admit of easy and convenient intercommunication among its inhabitants.

14. By statistics of disease, I mean returns of all sickness and accidents attended by the medical officers of districts; all such ailments as are medically relieved in those noble

* Essays on State Medicine, pp. 334-5.

† Where any sub-district necessarily includes persons living in very different localities, the vital statistics of those places should be separately returned.

hospitals and charitable dispensaries, which succour more than half of the labouring classes of England; all sickness occurring among bodies of workmen in public employ, or in legally established clubs and provident societies. Such a registration should have special regard to the causation of disease, and to its relation with residence and occupation. And this invaluable mass of information, now lost for want of collection, should be registered by the same machinery as that employed for registering the births and deaths of the people.

Meteorological observations, and the varying physical conditions of the animal and vegetable kingdoms, should be concurrently recorded in each superior registration district. And these combined observations and records should be published periodically in each locality for the instruction of its inhabitants; for as these become better informed on the various circumstances which affect their physical well-being, prejudices will subside, habits and manners will improve, opposition to improvements will cease, and local councils will become more useful and effective.

15. A second deduction from my preceding argument is, that the law should no longer confer imperfectly defined powers of a sanitary or reformatory nature upon two or more rival boards in the same place, yet with different and irreconcileable areas of jurisdiction. Now, as neither boards of guardians, nor town councils and other similar bodies, would be likely to consent to a general surrender of their sanitary functions to their rivals, I infer that *new* representative bodies ought to be instituted for the local administration of all matters affecting the public health and the physical condition of the people in every part of the kingdom, with larger jurisdictions than now belong to any of the corporate authorities which the legislature has partially and unsystematically empowered, and to be constituted, in great measure, of delegates from those established bodies. If each of the existing local boards and councils were fairly represented in a new superior court, as the district boards of London are in its metropolitan board, all reasonable objections to the proposed change might be removed.

I have elsewhere shown* how the philanthropic and scientific elements of control might be introduced into a higher kind of local administrative body; and, I would only add, that if these matters were directed in every district by better

* Essays on State Medicine, pp. 344-5.

constituted local councils, there would be less excuse for advocating any scheme of a centralising tendency, so repugnant to English notions.

16. Thirdly, as to the extent and form of the proposed jurisdictions. Instead of five hundred and eighty-seven provincial registration districts (I exclude those of the metropolis, both because that is now the subject of a new and somewhat doubtful experiment, and because London must always be dealt with separately and exceptionally), I recommend less than half that number. In other words, each superior district for the collection and registration of vital and sanitary statistics might contain, *on the average,* two or more parochial unions. Existing boundaries should, of course, be followed, unless some obvious advantage were to be gained by altering them; but wherever a correction of boundary might seem to be demanded, the physical geography of the locality should be carefully borne in mind; and, if possible, each parish or cluster of population should be included in that district, the principal town of which would be most easy of access. Special regard should be had to density of population. Where the *specific population* (as the French statists call it) might be under two hundred persons upon an English square mile (three acres and one-fifth to each person), a total population of 40,000 or 50,000 would suffice for the sanitary jurisdiction. Where it might exceed (say) four hundred upon a square mile (giving less than one acre and three-fifths to each person), a population of 80,000 would not be too many; while a much higher amount of population might be included in the case of a first-class town.

Further, every sanitary jurisdiction should be an exact aggregate of a sufficient number of small districts for medical visitation, which should be either identical with the registration subdistricts, or subdivisions of them. The boundaries of the existing union medical districts might be gradually and cautiously revised for the purpose.

17. Fourthly. Another object of great practical importance would be attained by the creation of the proposed larger sanitary jurisdictions. Every facility would then be afforded for the appointment of a superior class of officers of health. The superintendence of the registration of births and deaths; the collection of other statistics affecting life, health, and disease; the scientific observation and record of various natural phenomena; examinations and evidence in aid of forensic inquiries; the supervision of various preventive duties, as vaccination, measures against and during epidemics, etc.; the inspection of articles of food and medicine; all these and other duties, properly performed, would occupy the entire time of a skilled and experienced superintending officer,* who ought most certainly to be debarred from private professional engagements, and thus be rendered independent of those local influences which are known to be adverse to an unflinching and uncompromising discharge of public duty. Such an officer would be analogous to the *Kreis-physicus* of the German States, whose scientific reports and preventive duties are of great value, and would doubtless lead to more important practical results, if laid before an English community with its practical tendencies and its ample pecuniary resources.

18. The questions involved in the territorial distribution of the population are of the largest importance to society; but I must now close these remarks by a brief recapitulation.

I. The physical geography of the district, and the general character of its population, should be the main facts upon which any revision of the areas of sanitary inquiry and jurisdiction should be founded.

II. Areas for statistical returns should be co-extensive with those for sanitary management.

III. The extent of these areas should be large enough to provide satisfactorily for the amalgamation of existing smaller jurisdictions.

IV. The superior sanitary districts should also be large enough to secure, with economy, the appointment of a higher and more useful class of sanitary officers.

All these changes might be judiciously carried into effect, I believe, without any reckless or offensive sacrifice of existing interests, or any violation of justly established rights.

---

* It might be desirable to divide these functions among two or three officers, with different titles and qualifications. (Essays on State Medicine, pp. 50-1.)

